# Factors Associated With the Availability of Virtual Consultations in Primary Care Across 20 Countries: Cross-Sectional Study

**DOI:** 10.2196/65147

**Published:** 2025-03-19

**Authors:** Gabriele Kerr, Geva Greenfield, Edmond Li, Thomas Beaney, Benedict W J Hayhoe, Josip Car, Ana Clavería, Claire Collins, Gustavo Gusso, Robert D Hoffman, Geronimo Jimenez, Tuomas H Koskela, Liliana Laranjo, Heidrun Lingner, Ensieh Memarian, Katarzyna Nessler, Davorina Petek, Rosy Tsopra, Azeem Majeed, Ana Luisa Neves

**Affiliations:** 1 Department of Primary Care and Public Health Imperial College London London United Kingdom; 2 NIHR Applied Research Collaboration Northwest London London United Kingdom; 3 Institute of Global Health Innovation Department of Surgery and Cancer Imperial College London London United Kingdom; 4 School of Life Course and Population Sciences King’s College London London United Kingdom; 5 Primary Care Research Unit Servizo Galego de Saúde Vigo Spain; 6 I-Saúde Group Galicia Sur Health Research Institute Vigo Spain; 7 Irish College of General Practitioners Dublin Ireland; 8 Department of Public Health and Primary Care Ghent University Ghent Belgium; 9 Department of Internal Medicine Universidade de São Paulo São Paulo Brazil; 10 Department of Family Medicine Medical Faculty Tel Aviv University Tel Aviv Israel; 11 Department of Public Health and Primary Care Leiden University Leiden The Netherlands; 12 Faculty of Medicine and Health Technology Tampere University Tampere Finland; 13 The Wellbeing Services County of Pirkanmaa Tampere Finland; 14 Westmead Applied Research Centre Faculty of Medicine and Health University of Sydney Sydney Australia; 15 Center for Public Health and Healthcare Department of Medical Psychology Unit 5430 Hannover Medical School Hannover Germany; 16 Internal Medicine Research Group, Department of Clinical Sciences Faculty of Medicine Lund University Malmö Sweden; 17 Department of Family Medicine Jagiellonian University Medical College Krakow Poland; 18 Department of Family Medicine Faculty of Medicine University of Ljubljana Ljubljana Slovenia; 19 Centre de Recherche des Cordeliers Université Paris Cité & Sorbonne Université Paris France; 20 Assistance Publique-Hôpitaux de Paris Department of Medical Informatics Hôpital Européen Georges-Pompidou et Hôpital Necker-Enfants Malades Paris France

**Keywords:** digital health, primary care, telemedicine, virtual consultation, healthcare delivery, online consultation, primary care physician, upper-middle income, upper-middle income countries, high-income countries, online survey, chi-squared test, remote healthcare, video consultation, chat consultation, telephone consultations, digital technology, virtual care, teleconsultation, telehealth, remote consultation

## Abstract

**Background:**

Virtual consultations represent a notable change in health care delivery following the COVID-19 pandemic. Understanding the dynamics of virtual consultations is critical in assessing health care system resilience and adaptability in times of crisis.

**Objective:**

This study aimed to describe the availability and hours of use of telephone, video, and human chat consultations before and during the COVID-19 pandemic period, and identify factors associated with their availability.

**Methods:**

Primary care physicians (PCPs) from 20 upper-middle– and high-income countries completed a cross-sectional web-based survey in 2020. Factors associated with availability were investigated using chi-square tests and effect size (ES) estimates calculated using Cramer V.

**Results:**

A total of 1370 PCPs were included in this study (85.4% of the total sample of 1605). Telephone consultations were the most frequently available type of virtual consultations before and during the pandemic (73.1% and 90.4%, respectively). Significant increases in availability and use were observed during the pandemic for all the types of virtual consultations. The largest absolute increase in availability was observed for video consultations (39.5%), followed by telephone (17.3%) and chat (8.6%; all *P<*.001). The largest increase in use was observed for telephone consultations (+11 hours per week, *P*<.001). Digital maturity of the practice was weakly associated with availability of video consultations both before (ES 0.2) and during (ES 0.2) the pandemic (*P*<.001 for both), and with chat consultations before the pandemic only (ES 0.1, *P*=.001). Greater availability of video and chat consultations was found in PCPs who had completed digital health training, both before and during the pandemic (*P*<.001 for all). There was significant country-level variation in the use and availabilities of the technologies between both time periods. The association between country and the availability of telephone consultations changed from strong (ES 0.5, *P*<.001) to weak (ES 0.2, *P*=.03), while the relationship between country and video consultations changed from moderate (ES 0.3, *P*<.001) to strong (ES 0.5, *P*<.001).

**Conclusions:**

Our study demonstrates the transformative impact of the COVID-19 pandemic on the availability of virtual consultations globally, and how practice-level factors, predominantly digital maturity, digital health training, and country, were associated with the availability of virtual consultations. Further exploration of drivers of availability, particularly at the national level, is needed to ensure sustained and effective implementation of virtual consultations.

**International Registered Report Identifier (IRRID):**

RR2-10.2196/30099

## Introduction

The emergence of virtual consultations, defined as remote health care interactions facilitated by digital technologies, is a significant evolution in health care delivery. Telephone, video, and chat consultations may be more accessible than in-person appointments, as they offer rapid real-time communications with providers without a need to travel [1,2]. Despite these potential benefits, before the COVID-19 pandemic, these technologies, particularly telephone consultations, were steadily gaining traction, but had not reached widespread integration into most mainstream primary health care systems [3,4].

During the COVID-19 pandemic, virtual care became vital to the safe and efficient continuation of primary care delivery, when minimizing in-person encounters was essential to protect both health care staff and patients from the risk of infection [5,6]. Many health systems adopted some form of “virtual first” approach to primary health care provision [4]. The initial virtual encounter aimed to manage patients’ needs without in-person contact wherever possible while reserving “higher risk” face-to-face visits for those at greatest need, and where physical examination was deemed to be essential.

Throughout the pandemic, primary care physicians (PCPs) faced barriers in adopting and implementing virtual consultations, with potential consequences impacting the quality of care delivered to patients [2]. The ability of PCPs to effectively transition to virtual service delivery depends on multiple factors, including organizational and policy incentives, digital health infrastructure capacity and investment, cultural norms and attitudes, and the digital health literacy and skills of PCPs and patient populations [3,4,7-9]. These factors would act as barriers or drivers to differing degrees depending on the specific consultation technology, with likely fewer infrastructural or skill barriers for telephone consultations compared to video or chat consultations [10]. These factors may have resulted in variation in adoption and use of different virtual consulting technologies between PCPs and providers from different settings [2,4].

With growing demand for rapid and convenient access to primary care, alongside financial constraints requiring efficiency gains, virtual care appeared as an attractive solution to enhance patient accessibility [2]. Consequently, virtual consultations continue as a core component of health care delivery in many upper-middle– and high-income countries beyond the pandemic [4].

Examining the landscape of virtual health care technologies before and during the pandemic can help us better understand the magnitude of the transition to these new models of care. This transition highlights the investments made into the digital health capacity of primary care systems, carrying significant long-term implications for how care is delivered. However, how the availability and uptake of virtual consultations varied across PCPs from different settings, including different countries, is uncertain [11].

The aim of this study was to analyze access and use of virtual consultations before and during the pandemic, and factors associated with availability of virtual consulting technologies between PCPs from different settings. Specific aims include to analyze the availability and hours of use of telephone, video and chat consultations before and during the COVID-19 pandemic, and to identify factors associated with their availability.

## Methods

### Study Design

This study used data from a cross-sectional web-based questionnaire completed by PCPs of 20 upper-middle- and high-income countries (Australia, Brazil, Canada, Chile, Colombia, Croatia, Finland, France, Germany, Ireland, Israel, Italy, Poland, Portugal, Spain, Slovenia, Sweden, Türkiye, the United Kingdom, and the United States). The questionnaire was designed and administered by the inSIGHT Research Group, a consortium of academic primary care researchers from the 20 countries previously listed. The study adheres to the STROBE (STrengthening the Reporting of OBservational studies in Epidemiology) guidelines for reporting observational studies [12].

### Data Collection

Participants were eligible if they were practicing PCPs in 1 of the 20 countries listed above, between March and September 2020. The study was conducted between June and September 2020. National leads in each country invited PCPs through their formal organizations or personal networks through email or social media (ie, Facebook and Twitter [subsequently rebranded X]). The questionnaire was hosted on Qualtrics (Silver Lake) and was available in English, French, German, Italian, Spanish, and Portuguese. A complete description of the study protocol, including the full questionnaire and power analyses, has been previously published [13]. Sections of the questionnaire relevant to this study are included in pages 2-5 in Multimedia Appendix 1.

### Study Variables

Participants were asked to answer whether chat (ie, using a text-based messaging system), telephone or video consultations were available in their practice before or during the COVID-19 pandemic (from March 11, 2020). Respondents ticked a box for each period considered (ie, before or during the COVID-19 pandemic) to indicate a technology was available in that period.

Respondents were subsequently asked how many hours they spent per week on each type of consultation in each time period. Before analysis, hours per week spent on each of the 3 virtual consultation technologies were cleaned to remove answers of ≥100 hours per week. A response of >0 hours spent on a technology was considered evidence for the technology being available. This study includes PCPs who responded to at least 1 question on the availability or hours of use of virtual consultation technologies.

Predictor variables included country, urbanicity (rural, mixed, and urban), and practice digital maturity. Practice digital maturity was assessed using the digital maturity framework developed by Flott et al [14], which considers the 6 dimensions of usage, resources, and abilities (organizational and individual), interoperability, general evaluation methodology, and impact. PCPs could agree or disagree with 6 statements about their practice’s digital maturity, corresponding with the 6 dimensions. A digital maturity score was calculated for each PCP by granting 1 point for each statement with which the PCP indicated agreement, giving a possible range of 0 to 6 where a score of 6 indicates high digital maturity. PCPs were also asked whether they have completed training on digital technologies before or during the COVID-19 pandemic.

### Statistical Analysis

The total number of hours spent per week before and during the pandemic on virtual consultation technologies was calculated for PCPs who reported availability of at least one of the technologies in the period. For PCPs who reported the technology as available in both time periods, the number of hours spent by PCPs on each technology before and during the COVID-19 pandemic were compared using paired Wilcoxon signed rank tests, and the relationship between the predictors and change in hours of use of each technology was investigated using univariable linear regression models.

McNemar tests were conducted to compare availability of each technology before and during the COVID-19 pandemic period. Absolute differences in the percentage of PCPs with each technology available in each time period were described. Plots were created to visualize changes in technology availability and hours of use by country of PCP employment.

Cramer V was calculated to estimate the effect size (ES) of practice factors upon the variation in the availability of digital technologies before and during the COVID-19 pandemic. Cramer V estimates of ES to describe the strength of association between the predictors and outcomes were categorized as weak (0-0.29), moderate (0.3-0.49), or strong (≥0.5). The change in percentage of PCPs with each technology available was visualized by country. *P* values for statistical tests were adjusted for multiple comparisons using the Holm-Bonferroni method [15]. All analyses were performed in R (version 4.3; R Foundation for Statistical Computing) [16], and a significance level of .05 was used throughout.

### Ethical Considerations

Ethical approval was granted by the UK Imperial College Research Ethics Committee (reference 20IC5956), which oversees health-related research with human participants. Survey participants gave their written informed consent to participate in the study. Data collected were anonymous and no compensation was given for participation in the survey.

## Results

### Overview

This study includes responses from 1370 PCPs who responded to at least 1 question on the availability and/or hours of use of virtual consultation technologies, representing 85.4% of the total sample of 1605 (Table 1).

**Table 1:** Characteristics of the 1370 surveyed primary care physicians.

Characteristic		Primary care physicians, n (%)
**Age category (years)**		
	>30	79 (5.8)
	30-39	434 (31.7)
	40-49	356 (26)
	50-59	289 (21.1)
	60-69	192 (14)
	70+	16 (1.2)
	Prefer not to answer	4 (0.3)
	Missing	0 (0)
**Gender**		
	Female	827 (60.4)
	Male	535 (39.1)
	Other	1 (0.1)
	Prefer not to answer	7 (0.5)
	Missing	0 (0)
**Urbanicity**		
	Mixed	307 (22.4)
	Rural	211 (15.4)
	Urban	852 (62.2)
	Missing	0 (0)
**Years of PCP^a^ Experience (years)**		
	<5	265 (19.3)
	5-10	295 (21.5)
	10-15	210 (15.3)
	15-20	156 (11.4)
	>20	444 (32.4)
	Missing	0 (0)
**Digital maturity score**		
	0	108 (7.9)
	1	112 (8.2)
	2	130 (9.5)
	3	249 (18.2)
	4	268 (19.6)
	5	234 (17.1)
	6	269 (19.6)
**Country of PCP employment**		
	Australia	69 (5)
	Brazil	48 (3.5)
	Canada	47 (3.4)
	Chile	52 (3.8)
	Colombia	60 (4.4)
	Croatia	55 (4)
	Finland	43 (3.1)
	France	56 (4.1)
	Germany	46 (3.4)
	Ireland	237 (17.3)
	Israel	65 (4.7)
	Italy	89 (6.5)
	Poland	49 (3.6)
	Portugal	77 (5.6)
	Slovenia	66 (4.8)
	Spain	85 (6.2)
	Sweden	67 (4.9)
	Turkey	51 (3.7)
	United Kingdom	55 (4)
	United States	53 (3.9)
	Missing	0 (0)

^a^PCP: primary care physician.

The majority (827/1370, 60.4%) of the respondents were female and 57.6% (790/1370) were aged between 30-49 years. Almost a third (444/1370, 32.4%) of the respondents had clinical experience of more than 20 years. PCPs spent a median of 36 (IQR 28-40) hours on clinical work per week. The highest proportion of the respondents (852/1370, 62.2%) worked in practices based in urban areas. The median digital maturity score of their practices as reported by PCPs was 4 (IQR 2-5). Training on digital-first technologies was undertaken by (312/1370, 22.8%) PCPs before the pandemic and by (375/1370, 27.4%) PCPs during the pandemic period. A breakdown of PCP characteristics by country is available in Tables S1 and S2 in Multimedia Appendix 1.

### Use of Digital Technologies

PCPs reported spending a median of 3 hours per week using these tools (IQR 1-5), increasing to 15 (IQR 8-25) during the pandemic period (*P*<.001). Hours spent per week on specific technologies are shown in Table 2.

The average number of hours per week spent on each type of virtual consultation increased during the pandemic (Table 2). The greatest change was observed for time spent on telephone consultations (+11.0 hours/week, *P*<.001), on which 91.8% of PCPs reported spending increased time.

**Figure 1:**
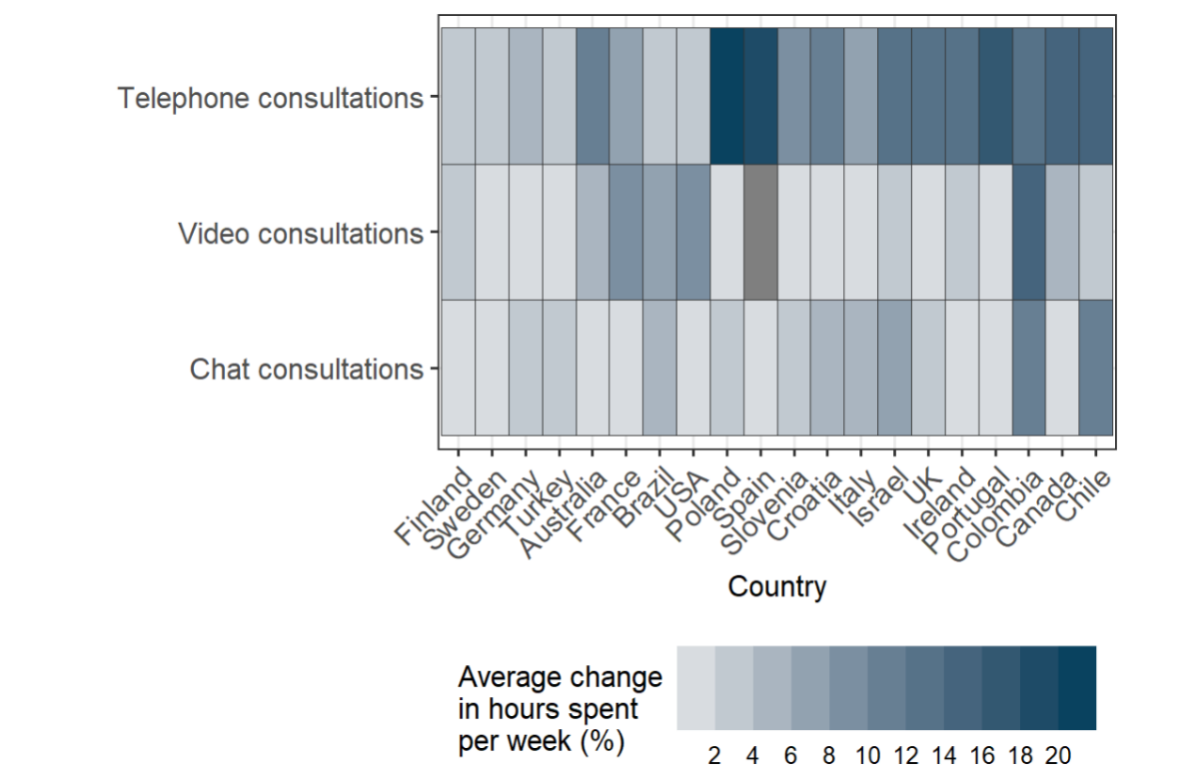
Mean change in hours spent per week on virtual consultation technologies by primary care physicians during versus before the COVID-19 pandemic period, by country of primary care physician employment. The COVID-19 pandemic period was defined as the period from March 11, 2020, onwards to the date of survey completion by the primary care physician (between June and September 2020). Grey cells indicate where no data was available for change in hours of use.

Country of PCP employment was associated with changes in hours spent per week on telephone (*R*^2^=0.2, *P*<.001) and chat consultations (*R*^2^=0.1, *P*=.001), but not with changes in hours spent on video consultations (*R*^2^=0.1, *P*=.73). The increase in hours spent per week on telephone consultations was largely driven by PCPs from Poland, Spain, Canada, Chile, and Portugal, who spent more than 15 additional hours per week on telephone consultations during the COVID-19 pandemic compared with before (Figure 1).

No association was found between changes in hours of use of any of the 3 virtual consultation technologies and any of practice digital maturity score, training, or urbanicity (Figure S1 in Multimedia Appendix 1).

**Table 2:** Average change in hours spent on virtual consultations by primary care physicians before and during the COVID-19 pandemic period, amongst primary care physicians who had the technology available in both time periods. The COVID-19 pandemic period was defined as the period from March 11, 2020 onwards to the date of survey completion by the primary care physicians (between June and September 2020).

Technology	Denominator	Mean hours spent per week before the pandemic	Mean hours spent per week during the pandemic	Mean difference in hours, mean (SD)^a^	*P* values^b^
Telephone consultations	883	3.8	14.2	+11 (10.7)	<.0001
Video consultations	127	1.3	4.3	+4.5 (7.3)	<.0001
Chat consultations (ie, using a messaging system)	365	2.4	5.3	+3.4 (6.7)	<.0001

^a^The mean difference describes the mean of the change in hours spent by each primary care physician on the technology.

^b^*P* values correspond to 2-sample Wilcoxon tests.

### Availability of Virtual Consultations Before and During the COVID-19 Pandemic

Before the pandemic, telephone consultations were the most frequently available virtual consultation technology (73.1%, 1002), followed by chat consultations (33.7%, 462) and video consultations (12.8%, 176). During the pandemic, telephone consultations remained the most frequently available solution (90.3%, 1238), followed by video consultations (52.3%, 717) and chat consultations (42.3%, 580).

**Figure 2:**
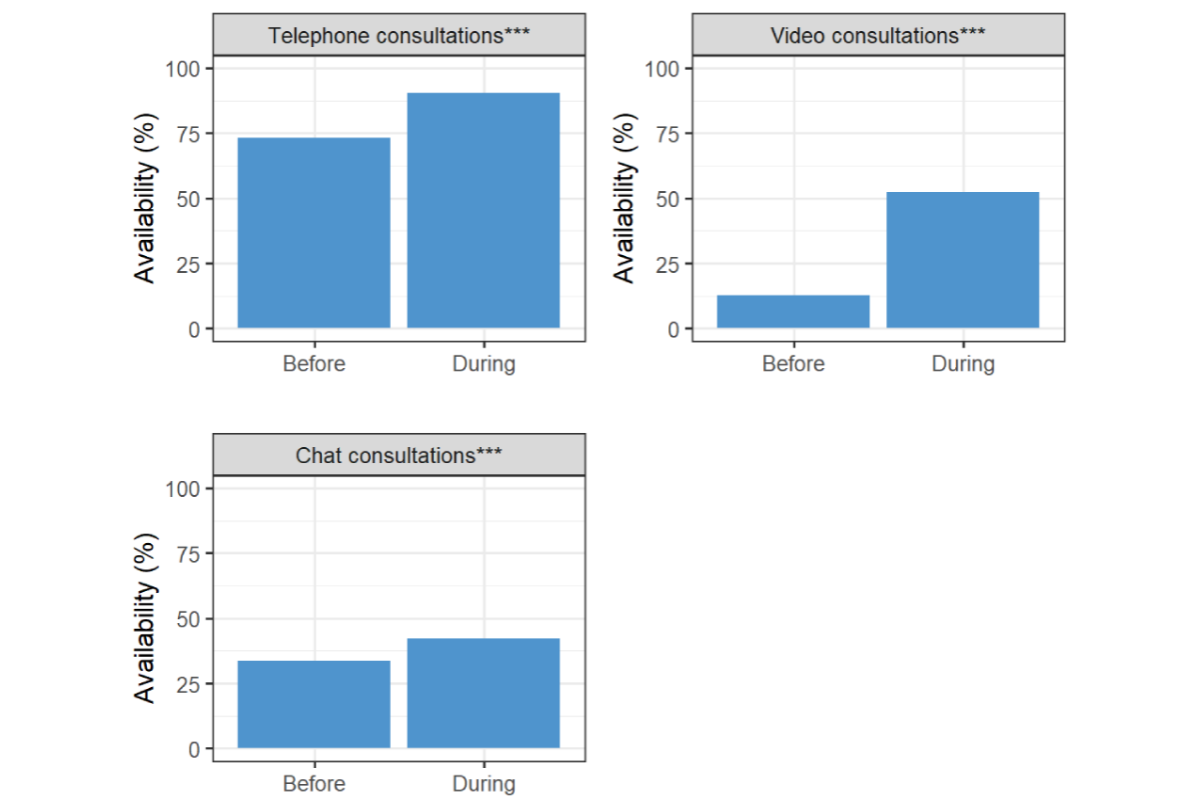
Percentages of primary care physicians who reported virtual consultation technologies as available to them before versus during the COVID-19 pandemic period. The COVID-19 pandemic period was defined as the period from March 11, 2020, to the date of survey completion by the PCP (between June and September 2020). ***: adjusted *P* values <.001.

Statistically significant increases were observed for all types of virtual consultation during the pandemic. During the pandemic, telephone consultations remained the most commonly available technology (90.4%, 1238; Figure 2). The largest absolute increase in availability was observed for video consultations (+39.5%, *P*<.001; Figure 2; Table S3 in Multimedia Appendix 1).

Of the 365 PCPs who lacked access to telephone consultations before the COVID-19 pandemic, 87.7% gained availability to this type of virtual consultation during the crisis. Correspondingly, 49.3% (n=584), and 21.8% (n=196) of PCPs who previously did not have access to video consultations, or chat consultations, respectively, reported gaining access to these technologies during the COVID-19 pandemic period.

### Factors Associated With Availability

Before the COVID-19 pandemic, practice urbanicity was weakly associated with availability of telephone consultations (ES 0.1, *P*<.001). Digital health training was weakly associated with availability of video (*P*<.001) and chat consultations (*P*=.04). Digital maturity score was weakly associated with increased availability of video consultations (*P*<.001) and chat consultations (*P*=.001). There was moderate to strong association between the country and availability of each of the technologies (Figure 2; ES range: 0.3-0.5, *P*<.001 for all).

During the pandemic, significant associations remained between availability and country, digital health training, and digital maturity score (Figure 2). Country persisted as significantly associated with the availability of chat consultations (*P*<.001), video consultations (*P*<.001), and telephone consultations (*P*=.03). Digital maturity score remained only weakly associated with the availability of video consultations (*P*<.001), but not with telephone or chat consultations. Digital health training was weakly associated with the availability of video (as observed before the pandemic), but also with chat consultations (*P*<.001 for both). Practice digital maturity score was no longer associated with chat consultations during the pandemic.

The strength of univariable associations between the availability of the technologies and the predictors differed before and during the COVID-19 pandemic. The strength of the associations between the country and telephone consultations decreased from strong to weak between the 2 time periods. In contrast, the strength of association between video consultations and country increased from moderate to strong.

A detailed overview of the nature of such associations is provided below.

#### Country Variations

**Figure 3:**
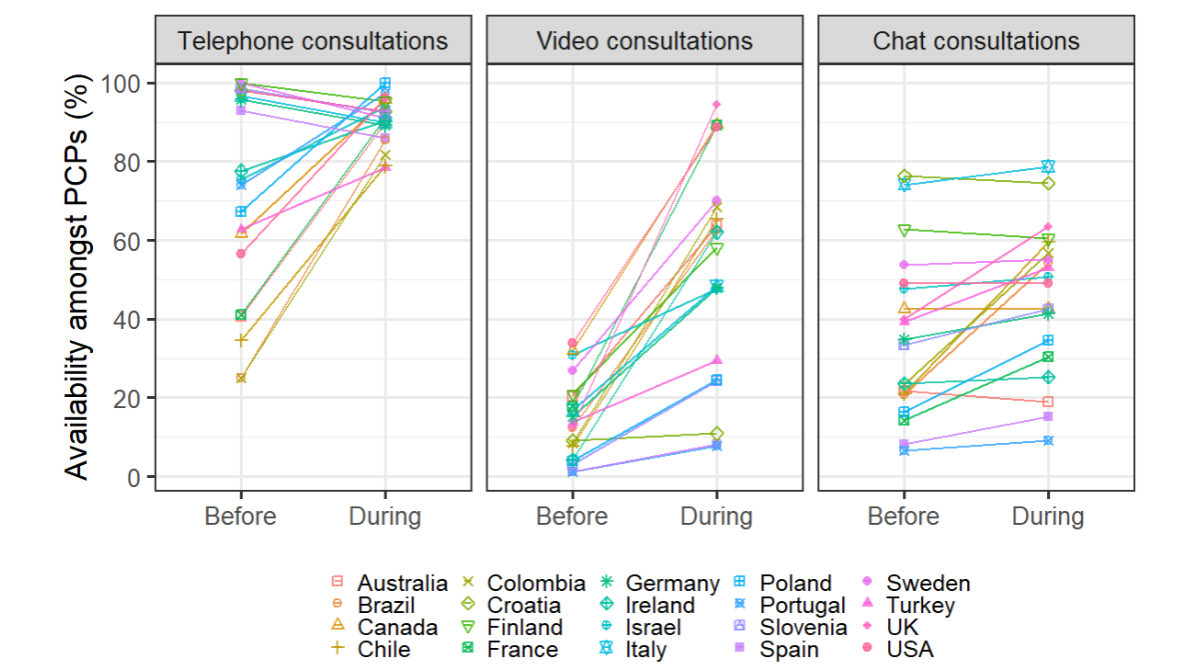
Absolute difference in percentage of primary care physicians from each country reporting the technology as available to them before versus during the COVID-19 pandemic. The COVID-19 pandemic period was defined as the period from March 11, 2020, to the date of survey completion by the primary care physician (between June and September 2020). PCP: primary care physician.

Availability of chat consultations varied greatly by country for both time periods, ranging from 9% to 78.7% for before the COVID-19 pandemic, and 6.5%-75.4% during the COVID-19 pandemic. Most countries showed only small changes in availability of chat consultations from before to during the COVID-19 pandemic (Figure 3). The largest changes in availability were observed for PCPs from Chile (+38.5%), Colombia (+33.3%), Brazil (+33.3%), and the United Kingdom (+23.6%). Changes in availability of <10% were observed for PCPs from 13 of the 20 countries surveyed.

**Figure 4:**
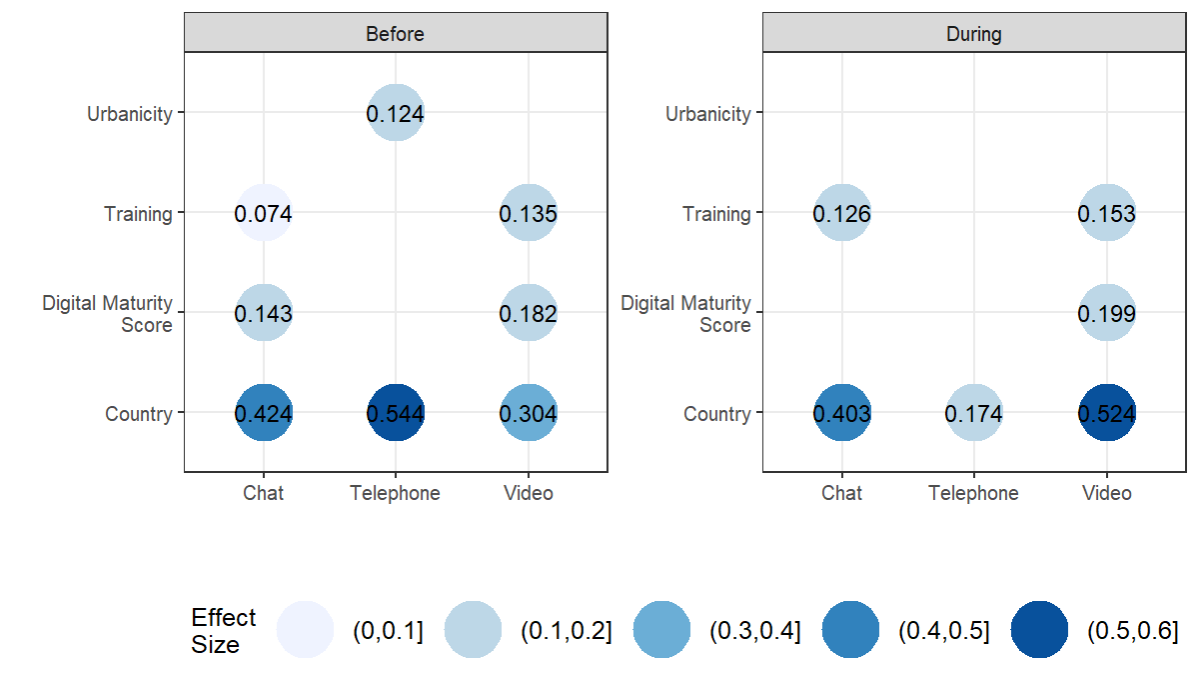
Effect size of primary care physician and practice factors on the reported availability of digital consultation technologies before and during the COVID-19 pandemic. The COVID-19 pandemic period was defined as the period from March 11, 2020, to the date of survey completion by the primary care physician (between June and September 2020). Effect sizes correspond to Cramer V measures of association; larger effect sizes indicate a stronger relationship between the predictor and availability. Estimates for nonsignificant relationships are not shown.

There was less variation in availability of telephone consultations between countries during than before the COVID-19 pandemic period (Figure 4). Before the pandemic, availability of telephone consultations across countries ranged from 25% to 100%, while during the pandemic, availability ranged from 78.4% to 100%. There were distinct differences in change in telephone consultation availability by country. Average availability of telephone consultations decreased amongst PCPs from countries, which reported >90% availability of telephone consultations before the COVID-19 pandemic while increases in availability were observed for all other countries.

Availability of video consultations was low across all countries before the COVID-19 pandemic period, with less than 35% of PCPs from each country having them available (range 1.1%-33.4%). Availability of video consultations increased on average for PCPs from all countries, to differing degrees by country (Figure 4). The largest increases were observed for PCPs from the United Kingdom (+81.8%), followed by PCPs from France (+71.4%), Colombia (+60.0%), and Ireland (+57.8%).

#### Urbanicity

Before the pandemic, the percentage of PCPs with telephone consultations available was highest amongst those from rural practices (83.4%), compared with mixed (77.5%) or urban settings (69%). This difference by practice urbanicity did not persist into the pandemic period. Availabilities of video and chat consultations were similar across PCPs from urban, mixed, and rural settings in both time periods.

#### Digital Health Training

Higher availability of video consultations was reported amongst PCPs who had completed, versus never completed, training in digital-first technologies, both before (18% vs 9%, *P*<.001) and during (61.1% vs 45.8%, *P*<.001) the pandemic. PCPs who had completed training reported greater availability of chat consultations before (37.8% vs 30.7%, *P*=.04) and during (49.5% vs 37%, *P*<.001) the pandemic period.

#### Digital Maturity

Availability of video consultations before and during the pandemic was greater amongst PCPs from more digitally mature practices. Availability of chat consultations before the pandemic was highest in PCPs from practices with a digital maturity score of 6 (42.7%), followed by 4 (39.6%) and 1 (35.7%). After adjustment for multiple testing, there was no association detected between digital maturity and availability of chat consultations during the pandemic.

## Discussion

### Principal Findings

Telephone consultations were the most frequently available type of virtual consultations both before and during the pandemic (73.1% and 90.4%, respectively). Significant increases in availability during the pandemic were observed for all the types of virtual consultations, alongside significant increases in hours spent on every type of virtual consultation. The largest increase in availability was observed for video consultations, whereas, although a minority of countries did display large increases in availability of chat consultations (Figure 3), a smaller change emerged in availability of chat consultations overall.

Regarding the factors associated with availability, PCPs from rural practices reported greater availability of telephone consultations before the COVID-19 pandemic but this association did not persist in the pandemic. Practice digital maturity was significantly (although weakly) associated with the availability of video consultations both before and during the pandemic, and with chat consultations before the pandemic only. Digital health training was weakly associated with the availability of both video and chat consultations, both before and during the pandemic.

There was significant country-level variation in the hours of use and availabilities of the technologies between both time periods (Figures 1 and 3). The association between country and the availability of telephone consultations changed from strong to weak, while the relationship between country and video consultations changed from moderate to strong. There was similarly strong country-level variation in availability of chat consultations in both periods.

### Comparison With Previous Work

Telephone consultations were the most frequently available and used virtual consultation modality, increasing during the pandemic compared with before. Their higher use and uptake were likely driven by their lower resource requirements and maintenance costs compared with video and chat consultations [7,10]. In addition, telephones are readily available to most patients and telephone consultations were already widely used in primary care in many places (Figure 3) [4], reducing the need for additional infrastructure or training. Supporting this, telephone consultation availability was independent of practicing digital maturity level or training in digital-first technologies, unlike video or chat technologies (Figure 2).

Before the pandemic, rural PCPs reported greater availability of telephone consultations compared with PCPs from urban or mixed settings. This is unsurprising, given the benefits of virtual consultations where geographic isolation can limit health care accessibility [17]. However, during the pandemic, availability of telephone consultations became similarly high amongst PCPs from rural, mixed, and urban settings, likely attributable to the need for social distancing and consequent adoption of telephone consultations in urban areas. Future research should address whether these changes persisted in the postpandemic period.

Smaller increases were apparent in the availability and use of chat consultations during the pandemic, compared with video. This may reflect specific implementation barriers for this type of virtual consultation, alongside their perception as an adjunct to, rather than as a replacement for, other consultation methods [18]. Previous UK research found that most online consultations required in-person or telephone follow-up [19,20]. There are safety considerations with chat consultations, including the challenges of identifying patient cues solely from written communication [21]. The proportionately greater increase in availability of video is likely explained by the ability to see the patient, which contributes substantially to the confidence of professionals in making a clinical assessment [22].

Adherence to data privacy regulations poses a particular challenge for implementing chat consultations, potentially discouraging uptake. Fulfilment of the legal obligation to record and store patient information can be difficult for chat consultations, necessitating PCPs to keep separate clinical records [23-25]. Most commonly used commercial messaging systems (eg, Telegram, iMessage, and WhatsApp) do not comply with health data privacy and security regulations [23,25]. Despite potential nonadherence to ethical or data privacy guidelines, commercial instant-messaging providers are widely used for clinical purposes by patients and health care staff [24,26-28].

Among the factors examined, the country had the strongest association with availability of virtual consultation technologies and was the only significant predictor of change in hours of use. The varied ability to transition to virtual service delivery between countries is likely attributable to various governance and infrastructural factors. Some countries have national long-term digitization goals for primary care, including strategies for virtual consultation adoption [29-31]. Coupled with guidelines on their effective and safe use [32,33], these would have facilitated greater adoption by PCPs. Countries also varied in their organizational and IT readiness to incorporate new consultation technologies into existing operations [9]. In the case of video and chat, regional variation in the availability of suitable platforms, internet coverage, and smart devices may have affected the feasibility of these consultations, contributing to a digital divide [10,31,34]. Implementation of video consultations in some countries was impeded by the need to update national health data regulations [3] and reimbursement policies [4,7,17,33]. It would be a valuable area of future work to map systems-level characteristics and explore associations between them and availability and use of virtual consultations.

Country-level variation in telephone consultation availability reduced during the pandemic compared with before, while the variation for video consultations increased (Figures 2 and 4). This indicates that the COVID-19 pandemic amplified discrepancies in barriers and facilitators of video consultation implementation between countries. Before the COVID-19 pandemic, video consultations were in the earlier stages of adoption in many countries, whereas telephone consultations were already widely available (Figure 3) and easier to scale up, for reasons previously stated [4]. Many countries continue to lack strategies for interoperability or digital education [29], likely contributing to the low adoption of video consultations post the pandemic [9]. Future research should investigate whether there have been sustained country-level differences in video consultation implementation beyond the pandemic.

### Strengths and Limitations

A primary strength of our study is the large number of PCPs surveyed from 20 countries, which included a mix of urban and rural settings, during a critical transition period for primary care service delivery. However, the findings must be interpreted in light of some accompanying weaknesses. The generalizability of the study’s findings may be limited by the reduced representativeness introduced by use of convenience sampling. Convenience sampling may introduce some self-selection bias for PCPs who hold stronger views about the research topic and are more vocal in sharing their experiences. Use of an anonymous web-based survey, disseminated by email and social media, prevented the identification of whether multiple respondents were employed at the same organization. In addition, due to the cross-sectional nature of the survey, we cannot establish directionality in the associations detected and causality should not be inferred. Nonetheless, these limitations are inherent to most survey-based studies and should not detract from the value of our findings.

In addition, the survey was not available in all the languages spoken by the countries surveyed which possibly excluded some PCPs from participating or affected their interpretation of questions. The study did not consider the type or size of health care organizations. Smaller practices may have incurred greater difficulties in transitioning to virtual service delivery models, particularly those in lower income areas, due to facing higher operating costs [7,10]. Another limitation is that PCPs were surveyed exclusively from upper-middle and high-income countries, restricting the generalizability of the findings to health care systems of similar economic contexts.

Finally, there have likely been significant changes in the implementation and perceptions of digital health technologies since the survey administration in 2020. An examination of availability and use of these virtual consultation technologies in more recent years, including in postpandemic contexts, would be a valuable area of future work. Nevertheless, these findings reflect a critical period for understanding the adaptability of health care systems in times of crisis.

### Implications for Policy and Practice

Understanding the variations in the availability of virtual consultation technologies within and between countries is essential to ensure that their continued use does not impose additional barriers [35]. While the pandemic reduced country-level discrepancies in the availability of telephone consultations, a widening gap emerged with the availability of video consultations. Further investigation is needed to determine if these disparities reflect variations in patient, clinician, or health care organization preferences, or if they stem from digital capacity limitations.

To fully harness the potential of digital health innovations, health care providers must possess a robust understanding of their capabilities, limitations, and ethical implications. However, despite the finding of a positive relationship between training in digital first technologies and availability of chat and video consultations, less than a third (27.4%) of PCPs had completed such training. There is therefore a need for comprehensive digital health training for physicians, ensuring that they are equipped with the digital health literacy essential for delivering optimal patient care in the modern health care landscape [11].

As video consultations experienced the most significant rise, it is crucial to establish through further research whether this pattern persists in current practice, and whether this consultation modality offers substantial advantages beyond simply reducing in-person interactions. It is possible that video consultations served primarily as a tool for clinical risk mitigation during the pandemic; as restrictions on in-person appointments have stopped, the high use of video consultations may have declined [9]. This underlines the need for further studies to understand the postpandemic landscape.

### Conclusions

This study highlights the significant role the COVID-19 pandemic played in driving the global adoption of virtual consultations in primary care. The increased use of virtual consultation technologies during the COVID-19 pandemic underscores the flexibility of primary care systems to adapt rapidly to the constraints imposed by the pandemic. This shift enabled continued service delivery while minimizing exposure risks for both patients and health care staff.

This research identified practice-level factors, particularly the country of practice and practice digital maturity, and digital health training, as key factors associated with the availability of these technologies. Although the COVID-19 pandemic motivated increased usage of virtual consultations overall, it also revealed widened discrepancies between countries in their ability to implement video consultations. Systems-level research is necessary to identify the country-level facilitators and barriers toward implementation of video consultations, to ensure their continued use.

## Appendix

Multimedia Appendix 1 Supplementary tables and figures.

## Abbreviations

ES: effect size
PCP: primary care physician
STROBE: STrengthening the Reporting of OBservational studies in Epidemiology

## Data Availability

The datasets generated and/or analyzed in this study are available upon reasonable request from the corresponding author.
