# Supplementary material for: Factors Associated With the Availability of Virtual Consultations in Primary Care Across 20 Countries: Cross-Sectional Study
Source: J Med Internet Res. 2025 Mar 19;27:e65147. doi: 10.2196/65147 (PMC11966075; doi:10.2196/65147)
Supplement: Multimedia Appendix 1 [file jmir_v27i1e65147_app1.docx]

# **Supplementary Appendix: Factors associated with the availability of virtual consultations in primary care across 20 countries: A cross-sectional study**

Gabriele Kerr^1,2^, Geva Greenfield^1,2^, Edmond Li^1,3^, Thomas Beaney^1^, Benedict WJ Hayhoe^1,2^, Josip Car^1,4^, Ana Clavería^5,6^, Claire Collins^7,8^, Gustavo Gusso^9^, Robert D Hoffman^10^, Geronimo Jimenez^11^, Tuomas H. Koskela^12^, Liliana Laranjo^13^, Heidrun Lingner^14^, Ensieh Memarian^15^, Katarzyna Nessler^16^, Davorina Petek^17^, Rosy Tsopra^18,19^, Azeem Majeed^1,2^, Ana Luisa Neves^1,2^

^1^Department of Primary Care and Public Health, Imperial College London, London, UK

^2^NIHR Applied Research Collaboration Northwest London, London, UK.

^3^Institute of Global Health Innovation, Department of Surgery and Cancer, Imperial College London, London, United Kingdom

^4^ School of Life Course and Population Sciences, King’s College London, London, UK

^5^Primary Care Research Unit, Vigo Health Area, Vigo, Spain

^6^I-Saúde Group. Galicia Sur Health Research Institute, Vigo, Spain

^7^Irish College of General Practitioners, Dublin, Ireland

^8^Deptartment of Public Health and Primary Care, Ghent University

^9^Department of Internal Medicine, Universidade de São Paulo (USP)

^10^ Department of Family Medicine, Medical Faculty, Tel Aviv University, Tel Aviv, Israel

^11^Department of Public Health and Primary Care, Leiden University Medical Center, Leiden, Netherlands

^12^Faculty of Medicine and Health Technology, Tampere University and The Wellbeing Services County of Pirkanmaa, Finland

^13^Westmead Applied Research Centre, Faculty of Medicine and Health, University of Sydney, Sydney, Australia

^14^Center for Public Health and Healthcare, Department of medical Psychology OE5430; German Center for Lung Research (DZL) / BREATH Hannover, Hannover Medical School, Hannover, Germany

^15^Department of Clinical Sciences, Malmö, Internal Medicine Research Group, Skåne University Hospital, Lund University, Malmo, Sweden.

^16^Department of Family Medicine, Jagiellonian University Medical College, Krakow, Poland

^17^Department of Family Medicine, Faculty of Medicine, University of Ljubljana, Ljubljana, Slovenia

^18^Université Paris Cité, Sorbonne Université, Inserm, Centre de Recherche des Cordeliers, F-75006 Paris, France

^19^Department of Medical Informatics, AP-HP, Hôpital Européen Georges-Pompidou et Hôpital Necker-Enfants Malades, F-75015 Paris, France

**Contents**

|  | **Page** |
| --- | --- |
| **Questionnaire** | 2 |
| **Tables** |  |
| **S1 Table:** Characteristics of GPs for countries A to I | 6 |
| **S2 Table:** Characteristics of GPs for countries I to U | 9 |
| **S3 Table:** Availability of virtual consultation technologies | 12 |
| **Figures** |  |
| **Supplementary Appendix Figure 1** | 13 |

**Questionnaire**

**Box S1:** Description of survey questions and possible answers.

| **Question** | **Answer Categories** |
| --- | --- |
| Please select your gender | Female  Male  Other  Prefer not to answer |
| Please select your age from the categories below: | Under 30  30-39  40-49  50-59  60-69  70+  Prefer not to answer |
| In which country do you currently practice? | Australia  Brazil  Canada  Chile  Colombia  Croatia  Finland  France  Germany  Ireland  Israel  Italy  Poland  Portugal  Slovenia  Spain  Sweden  Turkey  United Kingdom  United States |
| In which type of setting is your practice located? | Urban  Rural  Mixed |
| On average, how many hours of clinical work do you have per week? | [Free text input] |
| For how long have you been working as a General Practitioner/Family Doctor? | < 5 years  5 - 10 years  10 - 15 years  15 - 20 years  > 20 years |
| Are you involved in teaching activities? (i.e. teaching trainees or medical students, or affiliated to a university) | Yes  No  Prefer not to answer |
| In order to help us understand the digital maturity of your electronic health record system, please tell us how much you agree with the following statement:  Most healthcare providers in our practice use the digital system | Agree  Neutral  Disagree |
| In order to help us understand the digital maturity of your electronic health record system, please tell us how much you agree with the following statement:  Our organisation is ready to use the digital system correctly | Agree  Neutral  Disagree |
| In order to help us understand the digital maturity of your electronic health record system, please tell us how much you agree with the following statement:  We have the individual abilities needed to use the digital system correctly | Agree  Neutral  Disagree |
| In order to help us understand the digital maturity of your electronic health record system, please tell us how much you agree with the following statement:  Our digital system has the capability to communicate across services or with other systems | Agree  Neutral  Disagree |
| In order to help us understand the digital maturity of your electronic health record system, please tell us how much you agree with the following statement:  We have best practice digital maturity evaluation methods in place | Agree  Neutral  Disagree |
| In order to help us understand the digital maturity of your electronic health record system, please tell us how much you agree with the following statement:  Our system has a positive impact in terms of outcomes for patients, structure, process or finance | Agree  Neutral  Disagree |
| Please select which technologies were available in your practice, before and during the COVID-19 pandemic (from 11th March). Please select all which apply to these statements.  Telephone consultations | Available BEFORE the COVID-19 pandemic  Available DURING the COVID-19 pandemic  Not available |
| Please select which technologies were available in your practice, before and during the COVID-19 pandemic (from 11th March). Please select all which apply to these statements.  Video consultations | Available BEFORE the COVID-19 pandemic  Available DURING the COVID-19 pandemic  Not available |
| Please select which technologies were available in your practice, before and during the COVID-19 pandemic (from 11th March). Please select all which apply to these statements.  Chat consultations (ie. using a messaging system) | Available BEFORE the COVID-19 pandemic  Available DURING the COVID-19 pandemic  Not available |
| On average, how many hours per week did you usually spend in the following clinical  activities? Please enter a number for BEFORE and DURING and put 0 for technologies that were not available at that time.  Telephone consultations | BEFORE the COVID-19 pandemic: [free text input]  DURING the COVID-19 pandemic: [free text input] |
| On average, how many hours per week did you usually spend in the following clinical  activities? Please enter a number for BEFORE and DURING and put 0 for technologies that were not available at that time.  Video consultations | BEFORE the COVID-19 pandemic: [free text input]  DURING the COVID-19 pandemic: [free text input] |
| On average, how many hours per week did you usually spend in the following clinical  activities? Please enter a number for BEFORE and DURING and put 0 for technologies that were not available at that time.  Chat consultations | BEFORE the COVID-19 pandemic: [free text input]  DURING the COVID-19 pandemic: [free text input] |
| Digital-first models are defined as contacts with healthcare that happen firstly by an online means (i.e. telephone, video, website,... ) as opposed to the traditional face-to-face care. Were you offered any training on digital-first technologies? | Yes, BEFORE the COVID-19 pandemic  Yes, DURING the COVID-19 pandemic |

**Tables**

**S1 Table:** Characteristics of GPs for countries A to I.^1^

| Characteristic | Australia, N = 69 | Brazil, N = 48 | Canada, N = 47 | Chile, N = 52 | Colombia, N = 60 | Croatia, N = 55 | Finland, N = 43 | France, N = 56 | Germany, N = 46 | Ireland, N = 237 |
| --- | --- | --- | --- | --- | --- | --- | --- | --- | --- | --- |
| Age category |  |  |  |  |  |  |  |  |  |  |
| 30-39 | 13 (18.8) | 21 (43.8) | 13 (27.7) | 21 (40.4) | 38 (63.3) | 30 (54.5) | 13 (30.2) | 33 (58.9) | 7 (15.2) | 41 (17.3) |
| 40-49 | 20 (29.0) | 14 (29.2) | 10 (21.3) | 11 (21.2) | 13 (21.7) | 10 (18.2) | 13 (30.2) | 8 (14.3) | 12 (26.1) | 85 (35.9) |
| 50-59 | 26 (37.7) | 5 (10.4) | 12 (25.5) | 12 (23.1) | 4 (6.7) | 4 (7.3) | 8 (18.6) | 3 (5.4) | 20 (43.5) | 60 (25.3) |
| 60-69 | 9 (13.0) | 0 (0.0) | 7 (14.9) | 7 (13.5) | 1 (1.7) | 0 (0.0) | 4 (9.3) | 9 (16.1) | 5 (10.9) | 45 (19.0) |
| 70+ | 0 (0.0) | 0 (0.0) | 1 (2.1) | 0 (0.0) | 0 (0.0) | 0 (0.0) | 0 (0.0) | 0 (0.0) | 2 (4.3) | 6 (2.5) |
| Prefer not to answer | 1 (1.4) | 0 (0.0) | 0 (0.0) | 0 (0.0) | 0 (0.0) | 0 (0.0) | 0 (0.0) | 0 (0.0) | 0 (0.0) | 0 (0.0) |
| Under 30 | 0 (0.0) | 8 (16.7) | 4 (8.5) | 1 (1.9) | 4 (6.7) | 11 (20.0) | 5 (11.6) | 3 (5.4) | 0 (0.0) | 0 (0.0) |
| Gender |  |  |  |  |  |  |  |  |  |  |
| Female | 47 (68.1) | 25 (52.1) | 31 (66.0) | 31 (59.6) | 41 (68.3) | 45 (81.8) | 29 (67.4) | 35 (62.5) | 16 (34.8) | 147 (62.0) |
| Male | 22 (31.9) | 22 (45.8) | 16 (34.0) | 21 (40.4) | 19 (31.7) | 9 (16.4) | 14 (32.6) | 21 (37.5) | 30 (65.2) | 90 (38.0) |
| Other | 0 (0.0) | 0 (0.0) | 0 (0.0) | 0 (0.0) | 0 (0.0) | 0 (0.0) | 0 (0.0) | 0 (0.0) | 0 (0.0) | 0 (0.0) |
| Prefer not to answer | 0 (0.0) | 1 (2.1) | 0 (0.0) | 0 (0.0) | 0 (0.0) | 1 (1.8) | 0 (0.0) | 0 (0.0) | 0 (0.0) | 0 (0.0) |
| Urbanicity |  |  |  |  |  |  |  |  |  |  |
| Mixed | 8 (11.6) | 4 (8.3) | 3 (6.4) | 6 (11.5) | 12 (20.0) | 12 (21.8) | 10 (23.3) | 10 (17.9) | 14 (30.4) | 76 (32.1) |
| Rural | 13 (18.8) | 1 (2.1) | 1 (2.1) | 1 (1.9) | 1 (1.7) | 16 (29.1) | 9 (20.9) | 4 (7.1) | 17 (37.0) | 49 (20.7) |
| Urban | 48 (69.6) | 43 (89.6) | 43 (91.5) | 45 (86.5) | 47 (78.3) | 27 (49.1) | 24 (55.8) | 42 (75.0) | 15 (32.6) | 112 (47.3) |
| Experience |  |  |  |  |  |  |  |  |  |  |
| < 5 years | 4 (5.8) | 14 (29.2) | 9 (19.1) | 1 (1.9) | 31 (51.7) | 15 (27.3) | 7 (16.3) | 16 (28.6) | 6 (13.0) | 23 (9.7) |
| > 20 years | 31 (44.9) | 3 (6.3) | 18 (38.3) | 20 (38.5) | 5 (8.3) | 5 (9.1) | 13 (30.2) | 11 (19.6) | 18 (39.1) | 106 (44.7) |
| 10 - 15 years | 11 (15.9) | 11 (22.9) | 3 (6.4) | 9 (17.3) | 7 (11.7) | 9 (16.4) | 9 (20.9) | 3 (5.4) | 9 (19.6) | 35 (14.8) |
| 15 - 20 years | 12 (17.4) | 10 (20.8) | 7 (14.9) | 4 (7.7) | 3 (5.0) | 5 (9.1) | 3 (7.0) | 6 (10.7) | 7 (15.2) | 35 (14.8) |
| 5 - 10 years | 11 (15.9) | 10 (20.8) | 10 (21.3) | 18 (34.6) | 14 (23.3) | 21 (38.2) | 11 (25.6) | 20 (35.7) | 6 (13.0) | 38 (16.0) |
| Teaching activities |  |  |  |  |  |  |  |  |  |  |
| No | 20 (29.0) | 17 (35.4) | 9 (19.1) | 20 (38.5) | 17 (28.3) | 36 (65.5) | 9 (20.9) | 16 (28.6) | 12 (26.1) | 83 (35.0) |
| Prefer not to answer | 0 (0.0) | 0 (0.0) | 0 (0.0) | 0 (0.0) | 2 (3.3) | 0 (0.0) | 1 (2.3) | 0 (0.0) | 0 (0.0) | 1 (0.4) |
| Yes | 49 (71.0) | 31 (64.6) | 38 (80.9) | 32 (61.5) | 41 (68.3) | 19 (34.5) | 33 (76.7) | 40 (71.4) | 34 (73.9) | 153 (64.6) |
| Average hours of clinical work per week |  |  |  |  |  |  |  |  |  |  |
| Mean (SD) | 29 (12.5) | 33 (14.4) | 33 (13.7) | 39 (11.1) | 39 (17.2) | 36 (6.9) | 30 (10.3) | 42 (13.3) | 44 (9.9) | 33 (11.1) |
| Median (IQR) | 28 (20.0 - 40) | 38 (20.0 - 40) | 32 (22.0 - 40) | 44 (30.0 - 44) | 40 (33.8 - 48) | 40 (35.0 - 40) | 30 (20.0 - 38) | 42 (34.5 - 50) | 42 (40.0 - 50) | 35 (25.0 - 40) |
| (-1,28] | 35 (50.7) | 17 (35.4) | 18 (38.3) | 11 (21.2) | 13 (22.4) | 3 (5.6) | 14 (32.6) | 7 (12.5) | 1 (2.2) | 74 (31.2) |
| (28,36] | 13 (18.8) | 7 (14.6) | 14 (29.8) | 6 (11.5) | 4 (6.9) | 18 (33.3) | 11 (25.6) | 13 (23.2) | 7 (15.2) | 77 (32.5) |
| (36,40] | 14 (20.3) | 15 (31.3) | 6 (12.8) | 1 (1.9) | 14 (24.1) | 28 (51.9) | 16 (37.2) | 8 (14.3) | 12 (26.1) | 46 (19.4) |
| (40,100] | 7 (10.1) | 9 (18.8) | 9 (19.1) | 34 (65.4) | 27 (46.6) | 5 (9.3) | 2 (4.7) | 28 (50.0) | 26 (56.5) | 40 (16.9) |
| Missing | 0 (0.0) | 0 (0.0) | 0 (0.0) | 0 (0.0) | 2 (3.3) | 1 (1.7) | 0 (0.0) | 0 (0.0) | 0 (0.0) | 0 (0.0) |
| Duration of use |  |  |  |  |  |  |  |  |  |  |
| 2-5 years | 3 (4.5) | 15 (32.6) | 5 (10.9) | 8 (15.4) | 15 (25.0) | 10 (19.2) | 3 (7.0) | 12 (22.6) | 1 (3.1) | 12 (5.2) |
| 5-10 years | 11 (16.4) | 14 (30.4) | 19 (41.3) | 22 (42.3) | 17 (28.3) | 24 (46.2) | 8 (18.6) | 18 (34.0) | 7 (21.9) | 28 (12.1) |
| Before COVID-19 outbreak, but for less than 2 years | 4 (6.0) | 8 (17.4) | 2 (4.3) | 3 (5.8) | 5 (8.3) | 4 (7.7) | 0 (0.0) | 1 (1.9) | 1 (3.1) | 0 (0.0) |
| More than 10 years | 49 (73.1) | 7 (15.2) | 19 (41.3) | 19 (36.5) | 21 (35.0) | 14 (26.9) | 32 (74.4) | 22 (41.5) | 22 (68.8) | 190 (82.3) |
| Only after COVID-19 outbreak | 0 (0.0) | 2 (4.3) | 1 (2.2) | 0 (0.0) | 2 (3.3) | 0 (0.0) | 0 (0.0) | 0 (0.0) | 1 (3.1) | 1 (0.4) |
| Missing | 2 (3.0) | 2 (4.2) | 1 (2.1) | 0 (0.0) | 0 (0.0) | 3 (5.5) | 0 (0.0) | 3 (5.4) | 14 (30.4) | 6 (13.0) |
| Digital Maturity Score |  |  |  |  |  |  |  |  |  |  |
| 0 | 4 (5.8) | 6 (12.5) | 1 (2.1) | 2 (3.8) | 4 (6.7) | 5 (9.1) | 0 (0.0) | 5 (8.9) | 14 (30.4) | 7 (3.0) |
| 1 | 8 (11.6) | 10 (20.8) | 5 (10.6) | 6 (11.5) | 12 (20.0) | 5 (9.1) | 0 (0.0) | 1 (1.8) | 1 (2.2) | 1 (0.4) |
| 2 | 5 (7.2) | 11 (22.9) | 4 (8.5) | 12 (23.1) | 6 (10.0) | 11 (20.0) | 4 (9.3) | 6 (10.7) | 3 (6.5) | 6 (2.5) |
| 3 | 6 (8.7) | 10 (20.8) | 7 (14.9) | 12 (23.1) | 11 (18.3) | 7 (12.7) | 13 (30.2) | 13 (23.2) | 8 (17.4) | 31 (13.1) |
| 4 | 11 (15.9) | 5 (10.4) | 17 (36.2) | 13 (25.0) | 5 (8.3) | 14 (25.5) | 13 (30.2) | 15 (26.8) | 8 (17.4) | 38 (16.0) |
| 5 | 15 (21.7) | 5 (10.4) | 4 (8.5) | 2 (3.8) | 11 (18.3) | 8 (14.5) | 6 (14.0) | 9 (16.1) | 2 (4.3) | 73 (30.8) |
| 6 | 20 (29.0) | 1 (2.1) | 9 (19.1) | 5 (9.6) | 11 (18.3) | 5 (9.1) | 7 (16.3) | 7 (12.5) | 10 (21.7) | 81 (34.2) |

*^1^* Categorical data are shown as n (%)

**S2 Table:** Characteristics of GPs for countries I to U.^1^

| Characteristic | Israel, N = 65*^1^* | Italy, N = 89*^1^* | Poland, N = 49*^1^* | Portugal, N = 77*^1^* | Slovenia, N = 66*^1^* | Spain, N = 85*^1^* | Sweden, N = 67*^1^* | Türkiye, N = 51*^1^* | UK, N = 55*^1^* | USA, N = 53*^1^* |
| --- | --- | --- | --- | --- | --- | --- | --- | --- | --- | --- |
| Age category |  |  |  |  |  |  |  |  |  |  |
| 30-39 | 11 (16.9) | 25 (28.1) | 19 (38.8) | 49 (63.6) | 25 (37.9) | 12 (14.1) | 11 (16.4) | 19 (37.3) | 14 (25.5) | 19 (35.8) |
| 40-49 | 29 (44.6) | 7 (7.9) | 11 (22.4) | 10 (13.0) | 21 (31.8) | 13 (15.3) | 24 (35.8) | 12 (23.5) | 19 (34.5) | 14 (26.4) |
| 50-59 | 15 (23.1) | 16 (18.0) | 5 (10.2) | 3 (3.9) | 14 (21.2) | 37 (43.5) | 11 (16.4) | 8 (15.7) | 13 (23.6) | 13 (24.5) |
| 60-69 | 8 (12.3) | 39 (43.8) | 0 (0.0) | 10 (13.0) | 4 (6.1) | 19 (22.4) | 17 (25.4) | 0 (0.0) | 6 (10.9) | 2 (3.8) |
| 70+ | 2 (3.1) | 1 (1.1) | 1 (2.0) | 0 (0.0) | 0 (0.0) | 0 (0.0) | 2 (3.0) | 0 (0.0) | 0 (0.0) | 1 (1.9) |
| Prefer not to answer | 0 (0.0) | 0 (0.0) | 0 (0.0) | 0 (0.0) | 1 (1.5) | 0 (0.0) | 0 (0.0) | 0 (0.0) | 1 (1.8) | 1 (1.9) |
| Under 30 | 0 (0.0) | 1 (1.1) | 13 (26.5) | 5 (6.5) | 1 (1.5) | 4 (4.7) | 2 (3.0) | 12 (23.5) | 2 (3.6) | 3 (5.7) |
| Gender |  |  |  |  |  |  |  |  |  |  |
| Female | 44 (67.7) | 33 (37.1) | 33 (67.3) | 52 (67.5) | 50 (75.8) | 45 (52.9) | 29 (43.3) | 33 (64.7) | 29 (52.7) | 32 (60.4) |
| Male | 21 (32.3) | 56 (62.9) | 16 (32.7) | 25 (32.5) | 15 (22.7) | 39 (45.9) | 38 (56.7) | 18 (35.3) | 25 (45.5) | 18 (34.0) |
| Other | 0 (0.0) | 0 (0.0) | 0 (0.0) | 0 (0.0) | 1 (1.5) | 0 (0.0) | 0 (0.0) | 0 (0.0) | 0 (0.0) | 0 (0.0) |
| Prefer not to answer | 0 (0.0) | 0 (0.0) | 0 (0.0) | 0 (0.0) | 0 (0.0) | 1 (1.2) | 0 (0.0) | 0 (0.0) | 1 (1.8) | 3 (5.7) |
| Urbanicity |  |  |  |  |  |  |  |  |  |  |
| Mixed | 7 (10.8) | 19 (21.3) | 14 (28.6) | 18 (23.4) | 18 (27.3) | 23 (27.1) | 18 (26.9) | 12 (23.5) | 12 (21.8) | 11 (20.8) |
| Rural | 5 (7.7) | 16 (18.0) | 4 (8.2) | 7 (9.1) | 12 (18.2) | 20 (23.5) | 19 (28.4) | 4 (7.8) | 3 (5.5) | 9 (17.0) |
| Urban | 53 (81.5) | 54 (60.7) | 31 (63.3) | 52 (67.5) | 36 (54.5) | 42 (49.4) | 30 (44.8) | 35 (68.6) | 40 (72.7) | 33 (62.3) |
| Experience |  |  |  |  |  |  |  |  |  |  |
| < 5 years | 6 (9.2) | 25 (28.1) | 23 (46.9) | 16 (20.8) | 14 (21.2) | 10 (11.8) | 11 (16.4) | 18 (35.3) | 9 (16.4) | 7 (13.2) |
| > 20 years | 17 (26.2) | 50 (56.2) | 5 (10.2) | 13 (16.9) | 18 (27.3) | 50 (58.8) | 22 (32.8) | 10 (19.6) | 15 (27.3) | 14 (26.4) |
| 10 - 15 years | 17 (26.2) | 3 (3.4) | 5 (10.2) | 16 (20.8) | 10 (15.2) | 9 (10.6) | 8 (11.9) | 13 (25.5) | 11 (20.0) | 12 (22.6) |
| 15 - 20 years | 9 (13.8) | 6 (6.7) | 5 (10.2) | 4 (5.2) | 10 (15.2) | 10 (11.8) | 7 (10.4) | 2 (3.9) | 6 (10.9) | 5 (9.4) |
| 5 - 10 years | 16 (24.6) | 5 (5.6) | 11 (22.4) | 28 (36.4) | 14 (21.2) | 6 (7.1) | 19 (28.4) | 8 (15.7) | 14 (25.5) | 15 (28.3) |
| Teaching activities |  |  |  |  |  |  |  |  |  |  |
| No | 15 (23.1) | 38 (42.7) | 32 (65.3) | 22 (28.6) | 24 (36.4) | 16 (18.8) | 21 (31.3) | 20 (39.2) | 16 (29.1) | 15 (28.3) |
| Prefer not to answer | 1 (1.5) | 0 (0.0) | 0 (0.0) | 1 (1.3) | 1 (1.5) | 0 (0.0) | 0 (0.0) | 1 (2.0) | 0 (0.0) | 1 (1.9) |
| Yes | 49 (75.4) | 51 (57.3) | 17 (34.7) | 54 (70.1) | 41 (62.1) | 69 (81.2) | 46 (68.7) | 30 (58.8) | 39 (70.9) | 37 (69.8) |
| Average hours of clinical work per week |  |  |  |  |  |  |  |  |  |  |
| Mean (SD) | 29 (10.9) | 36 (12.3) | 28 (9.9) | 41 (9.5) | 39 (10.1) | 38 (9.2) | 31 (11.8) | 38 (8.4) | 29 (13.0) | 37 (16.8) |
| Median (IQR) | 30 (22.0 - 36) | 35 (30.0 - 40) | 25 (25.0 - 35) | 40 (40.0 - 45) | 40 (35.0 - 44) | 37 (35.0 - 40) | 35 (20.0 - 40) | 40 (40.0 - 40) | 30 (20.0 - 36) | 40 (30.0 - 50) |
| Hours of clinical work per week |  |  |  |  |  |  |  |  |  |  |
| (-1,28] | 30 (46.2) | 22 (24.7) | 25 (53.2) | 5 (6.6) | 6 (9.1) | 4 (4.7) | 23 (34.8) | 4 (7.8) | 23 (41.8) | 13 (24.5) |
| (28,36] | 20 (30.8) | 28 (31.5) | 12 (25.5) | 7 (9.2) | 19 (28.8) | 35 (41.2) | 13 (19.7) | 6 (11.8) | 20 (36.4) | 7 (13.2) |
| (36,40] | 10 (15.4) | 17 (19.1) | 6 (12.8) | 35 (46.1) | 23 (34.8) | 33 (38.8) | 26 (39.4) | 34 (66.7) | 6 (10.9) | 13 (24.5) |
| (40,100] | 5 (7.7) | 22 (24.7) | 4 (8.5) | 29 (38.2) | 18 (27.3) | 13 (15.3) | 4 (6.1) | 7 (13.7) | 6 (10.9) | 20 (37.7) |
| Missing | 0 (0.0) | 0 (0.0) | 2 (4.1) | 1 (1.3) | 0 (0.0) | 0 (0.0) | 1 (1.5) | 0 (0.0) | 0 (0.0) | 0 (0.0) |
| Duration of use |  |  |  |  |  |  |  |  |  |  |
| 2-5 years | 6 (9.2) | 17 (19.3) | 16 (37.2) | 6 (7.9) | 15 (30.6) | 4 (4.8) | 3 (4.6) | 12 (27.9) | 1 (1.8) | 7 (13.5) |
| 5-10 years | 16 (24.6) | 5 (5.7) | 9 (20.9) | 20 (26.3) | 13 (26.5) | 15 (18.1) | 9 (13.8) | 8 (18.6) | 8 (14.5) | 18 (34.6) |
| Before COVID-19 outbreak, but for less than 2 years | 3 (4.6) | 10 (11.4) | 12 (27.9) | 0 (0.0) | 11 (22.4) | 2 (2.4) | 1 (1.5) | 8 (18.6) | 3 (5.5) | 0 (0.0) |
| More than 10 years | 40 (61.5) | 56 (63.6) | 4 (9.3) | 50 (65.8) | 5 (10.2) | 62 (74.7) | 49 (75.4) | 15 (34.9) | 43 (78.2) | 27 (51.9) |
| Only after COVID-19 outbreak | 0 (0.0) | 0 (0.0) | 2 (4.7) | 0 (0.0) | 5 (10.2) | 0 (0.0) | 3 (4.6) | 0 (0.0) | 0 (0.0) | 0 (0.0) |
| Missing | 0 (0.0) | 1 (1.5) | 6 (12.2) | 1 (1.3) | 17 (25.8) | 2 (2.4) | 2 (3.0) | 8 (15.7) | 0 (0.0) | 1 (1.9) |
| Digital Maturity Score |  |  |  |  |  |  |  |  |  |  |
| 0 | 0 (0.0) | 3 (3.4) | 8 (16.3) | 1 (1.3) | 24 (36.4) | 6 (7.1) | 3 (4.5) | 13 (25.5) | 1 (1.8) | 1 (1.9) |
| 1 | 2 (3.1) | 8 (9.0) | 4 (8.2) | 13 (16.9) | 15 (22.7) | 2 (2.4) | 5 (7.5) | 10 (19.6) | 2 (3.6) | 2 (3.8) |
| 2 | 2 (3.1) | 9 (10.1) | 6 (12.2) | 7 (9.1) | 9 (13.6) | 12 (14.1) | 6 (9.0) | 5 (9.8) | 2 (3.6) | 4 (7.5) |
| 3 | 12 (18.5) | 23 (25.8) | 10 (20.4) | 18 (23.4) | 15 (22.7) | 17 (20.0) | 14 (20.9) | 8 (15.7) | 7 (12.7) | 7 (13.2) |
| 4 | 15 (23.1) | 22 (24.7) | 12 (24.5) | 22 (28.6) | 3 (4.5) | 18 (21.2) | 13 (19.4) | 4 (7.8) | 14 (25.5) | 6 (11.3) |
| 5 | 19 (29.2) | 13 (14.6) | 6 (12.2) | 8 (10.4) | 0 (0.0) | 17 (20.0) | 9 (13.4) | 5 (9.8) | 15 (27.3) | 7 (13.2) |
| 6 | 15 (23.1) | 11 (12.4) | 3 (6.1) | 8 (10.4) | 0 (0.0) | 13 (15.3) | 17 (25.4) | 6 (11.8) | 14 (25.5) | 26 (49.1) |

*^1^* Categorical data are shown as n (%)

**S3 Table:** Availability of virtual consultation technologies for N = 1,370 PCPs, before and during the COVID-19 pandemic, and associated statistic and p-values from McNemar tests.

| Technology | Missing (n, %)** | Available Before (n, %) | Available During (n, %) | Change (n, %) | Statistic | P value |
| --- | --- | --- | --- | --- | --- | --- |
| Telephone consultations | 3 (0.2) | 1,002 (73.1) | 1,238 (90.4) | 236 (+17.3) | 136.7 | <0.0001 |
| Chat consultations (i.e., using a messaging system) | 7 (0.5) | 462 (33.7) | 580 (42.3) | 118 (+8.6) | 50.0 | <0.0001 |
| Video consultations | 9 (0.7) | 176 (12.8) | 717 (52.3) | 541 (+39.5) | 465.1 | <0.0001 |

**Figures**


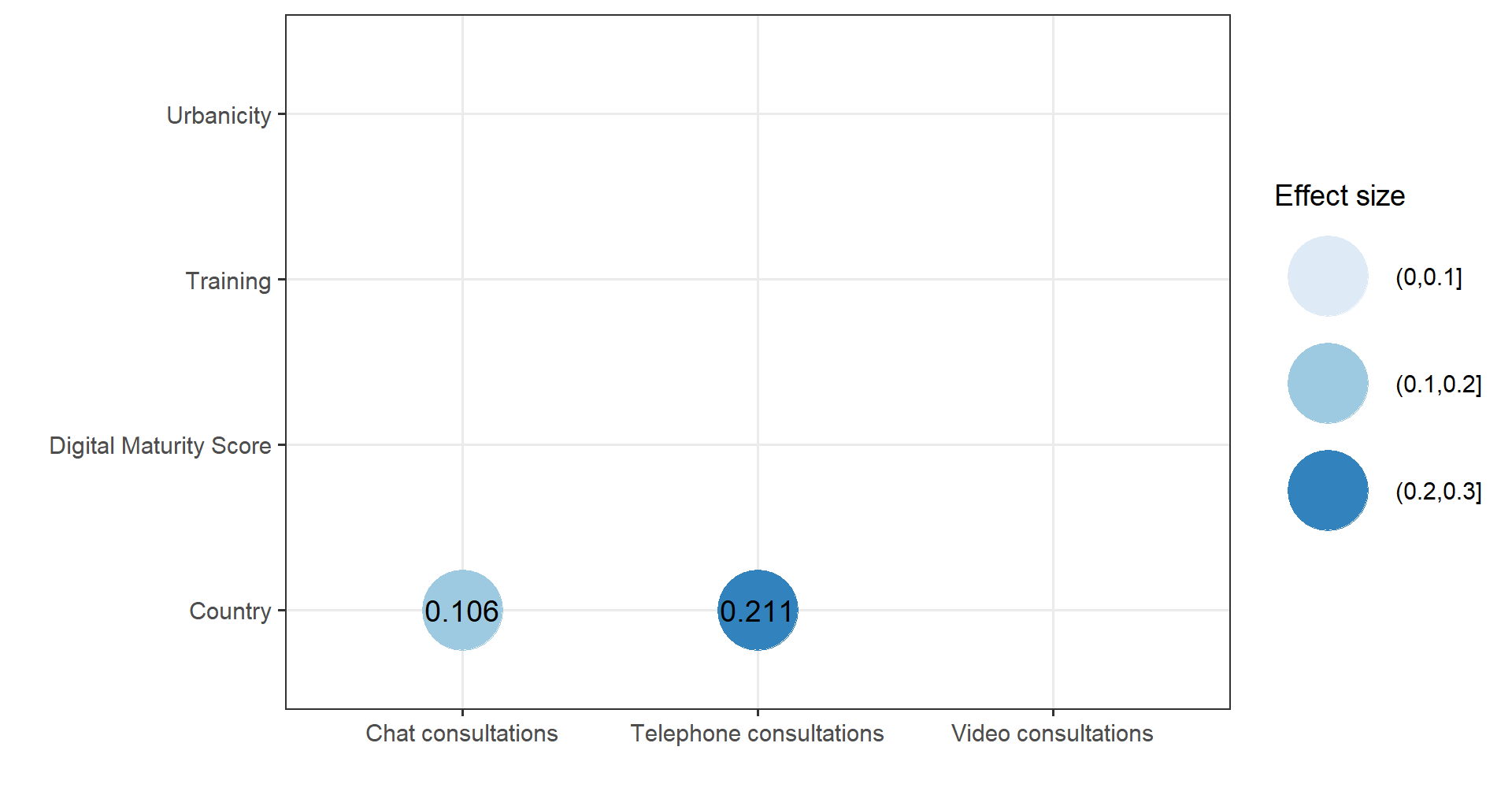


**Supplementary Appendix Figure 1 -** Effect size (R^2^) of practice and PCP characteristics on change in hours spent on virtual consultation technologies between before and during the COVID-19 pandemic. Non-statistically significant relationships are not shown.
